# Long noncoding RNA MALAT1 releases epigenetic silencing of HIV-1 replication by displacing the polycomb repressive complex 2 from binding to the LTR promoter

**DOI:** 10.1093/nar/gkz117

**Published:** 2019-02-21

**Authors:** Di Qu, Wei-Wei Sun, Li Li, Li Ma, Li Sun, Xia Jin, Taisheng Li, Wei Hou, Jian-Hua Wang

**Affiliations:** 1 CAS Key Laboratory of Molecular Virology and Immunology, Institut Pasteur of Shanghai, Chinese Academy of Sciences, Shanghai 200031, China; 2 University of Chinese Academy of Sciences, Beijing 100039, China; 3 School of Basic Medical Sciences, Wuhan University, Wuhan, Hubei 430070, China; 4 State Key Laboratory of Virology, Wuhan University, Wuhan, Hubei 430070, China; 5 Department of Infectious Diseases, Peking Union Medical College Hospital, Peking Union Medical College, Chinese Academy of Medical Sciences, Beijing 100730, China

## Abstract

Long noncoding RNAs (lncRNAs) may either repress or activate HIV-1 replication and latency; however, specific mechanisms for their action are not always clear. In HIV-1 infected CD4^+^ T cells, we performed RNA-Sequencing (RNA-Seq) analysis and discovered an up-regulation of MALAT1 (metastasis-associated lung adenocarcinoma transcript 1), an lncRNA previously described in cancer cells that associate with cancer pathogenesis. Moreover, we found that MALAT1 promoted HIV-1 transcription and infection, as its knockdown by CRISPR/Cas9 markedly reduced the HIV-1 long terminal repeat (LTR)-driven gene transcription and viral replication. Mechanistically, through an association with chromatin modulator polycomb repressive complex 2 (PRC2), MALAT1 detached the core component enhancer of zeste homolog 2 (EZH2) from binding with HIV-1 LTR promoter, and thus removed PRC2 complex-mediated methylation of histone H3 on lysine 27 (H3K27me3) and relieved epigenetic silencing of HIV-1 transcription. Moreover, the reactivation of HIV-1 stimulated with latency reversal agents (LRAs) induced MALAT1 expression in latently infected cells. Successful combination antiretroviral therapy (cART) was accompanied by significantly diminished MALAT1 expression in patients, suggesting a positive correlation of MALAT1 expression with HIV-1 replication. Our data have identified MALAT1 as a promoter of HIV-1 transcription, and suggested that MALAT1 may be targeted for the development of new therapeutics.

## INTRODUCTION

HIV-1 depends on host machineries for completing its life cycle (1–4). The identification of host factors that regulate HIV-1 replication may provide potential targets for the development of new drugs.

Long noncoding RNAs (LncRNAs) are a new class of host factors that attracted much attention recently. These are the most abundant type of noncoding RNAs, with more than 200 nucleotides in length, and they have been implicated in various physiological and pathological processes, such as epigenetic control of gene expression, chromatin organization, genomic imprinting, immune regulation, cell differentiation and development, viral pathogenesis and oncogenesis (5–13). Accumulating data have shown that lncRNAs either repress or activate HIV-1 replication and latency through regulating different cellular machineries. For instance, 7SK RNA is an abundant 331 nucleotides small nuclear RNAs that inhibits the cyclin-dependent kinase activity of P-TEFb (the positive transcription elongation factor) and represses gene transcription. The mechanism of its action is forming the small nuclear ribonucleoprotein complex (snRNP) in association with several proteins including the double-stranded RNA-binding protein HEXIM1 (hexamethylene bisacetamide induced protein 1) and HEXIM2, MEPCE (methyl-phosphate capping enzyme) and LARP7 (la ribonucleoprotein domain family member 7), and thus sequestering Cyclin T1/CDK9 in the 7SK RNP in a catalytic inactive form (14–20). Another LncRNA NEAT1 is an essential component of nuclear structure termed paraspeckle (21–23), which contains more than 30 nuclear proteins including RNA-binding proteins p54nrb (non-pou domain-containing octamer-binding protein), PSF (also known as splicing factor proline-glutamine rich) and Matrin3. NEAT1 is presumed to form the long-postulated nuclear compartment for storing HIV-1 Rev-dependent *cis*-acting instability element (INS)-containing RNAs (24,25). NEAT1 knockdown reduces the paraspeckle formation and enhances HIV-1 production through increasing the nucleus-to-cytoplasm export of HIV-1 INS-containing unspliced mRNAs (26). The activation of resting CD4^+^ T cells by phytohemagglutinin (PHA) could significantly reduce the NEAT1 expression and enhance viral replication (27). The lncRNA growth arrest-specific transcript 5 (GAS5) interacts with microRNA-873 to repress its HIV-1 replication promoting activity (28). The lncRNA NRON [noncoding repressor of nuclear factor of activated T-cells (NFAT)] inhibits HIV LTR-driven transcription through an association with the positive transcription factor NFAT, and thus preventing its nuclear translocation and subsequent binding with LTR. Intriguingly, viral Nef and Vpu proteins reduce and increase NRON expression, respectively (29). Moreover, NRON represses HIV-1 transcription and maintains viral latency by inducing the degradation of HIV-1 Tat protein (30). Unlike the abovementioned lncRNA species, uc002yug.2 is a 2564 base pair lncRNA, it enhances HIV-1 replication and reactivates HIV-1 from latency by promoting HIV-1 LTR-driven transcription. Mechanistically, uc002yug.2 upregulates the expression of Tat and downregulates the expression of transcription repressors RUNX (runt-related transcription factor)-1b and -1c by altering the splicing of RUNX1 pre-mRNA (31).

MALAT1 (metastasis-associated lung adenocarcinoma transcript 1) is a nuclear-enriched lncRNA that was originally discovered in metastatic carcinoma cells, and subsequently found to be markedly upregulated in many types of cancer (32), and thus it has been investigated as a prognostic biomarker and potential therapeutic target for metastatic cancers (5,32–38). Subsequently studies have found that MALAT1 is also abundantly expressed in noncancer cells, having high degree of conservation among mammals, and participating in multiple physiological and pathological processes (21,33,39–50). In particular, MALAT1 modulates gene transcription and alternative splicing of pre-mRNAs (12,33), having exclusive expression in nuclear and preferential location in the nuclear speckles (39,40), and having close interactions with several of the speckle-enriched proteins. Of note, MALAT1 localizes to chromatin sites containing active genes (51). It may influence gene expression by regulating the recruitment of specific chromatin modifiers or transcription factors to gene loci. For instance, MALAT1 regulates the localization of polycomb 2 protein (Pc2), a subunit of chromatin-modulating protein PRC-1. Upon serum stimulation, MALAT1 drove the translocation of unmethylated Pc2, along with several cell cycle associated proteins, from polycomb bodies to nuclear speckles, leading to the activation of growth-control gene program (41).

Notably, MALAT1 shows both physical and functional interactions with the PRC2 complex. Physical interaction between MALAT1 and both PRC2 subunits, the enhancer of zeste 2 (EZH2) and the suppressor of zeste 12 homolog (SUZ12), has been observed in T-cell lymphoma (52). Functionally, MALAT1 promotes EZH2 occupancy and increases H3K27 trimethylation level at Polycomb target loci, thereby enhances EZH2-mediated gene repression, cell invasion and migration of cancer cells (53–55). Alternatively, MALAT1 associates with SUZ12 or EZH2 to increase the expression of N-cadherin and reduce the expression of E-cadherin, and thus facilitates tumor malignancy (36,37).

The role of MALA1 in regulating HIV-1 infection is not completely understood. Some recent studies have shown that through mediating H3K27 trimethylation on LTR of HIV-1 provirus, the catalytic subunit EZH2 causes viral transcription silencing, and thus modulates the establishment and maintenance of HIV-1 latency (56–60). Clinically, cART-treated HIV-1-infected individuals have a significantly reduced MALA1 expression (61). The above studies imply that there is an association between MALAT1 and HIV-1 replication. To examine the molecular mechanisms on how MALAT1 regulates HIV-1 replication, we performed a series of molecular, biochemical and virological experiments and demonstrated that MALAT1 promotes HIV transcription and infection, via its association with PRC2 core component EZH2, thus preventing EZH2 from binding to HIV-1 LTR and disabling PRC2 complex mediated epigenetic silencing of HIV-1 genes. The identification of MALA1 as a promoter of HIV-1 transcription suggests it may be examined as a therapeutic target.

## MATERIALS AND METHODS

### Ethics statement

The usage of human cells and the related methods and experimental protocols have been approved by the Medical Ethics Review Committee of Institut Pasteur of Shanghai, Chinese Academy of Sciences, and the Medical Ethics Review Committee of Peking Union Medical College Hospital. All experiments were performed in accordance with relevant national guidelines and regulations.

### Cells

Peripheral blood mononuclear cells (PBMCs) from health donors were purchased from Shanghai Blood Center, Shanghai, China. CD4^+^ T cells were purified from PBMCs using anti-CD4 antibody-coated magnetic beads (Miltenyi Biotec). Resting CD4^+^ T cells were activated by treating with 5 μg/ml phytohaemagglutinin-P (PHA-P) (Sigma) for 3 days in the presence of IL-2 (20 IU/ml). The HEK293T cells were kindly provided by Dr Li Wu (The Ohio State University, USA). ACH2 is a clone of HIV-1 latently infected CD4^+^ CEM cell that contains a single copy of proviral DNA per cell (62–64) (provided by Dr Shi-Bo Jiang, Fudan University, Shanghai, China). HIV-1 latently infected Jurkat T cell (C11 clone) was provided by Dr Huan-Zhang Zhu (Fudan University, Shanghai, China). CD4^+^ T-lymphocyte cell line Hut/CCR5, Jurkat T cell and ACH2 were grown in RPMI 1640 medium supplemented with 10% fetal bovine serum (Gibco), 100 U/ml penicillin and 100 μg/ml of streptomycin (Invitrogen) at 37°C under 5% CO_2_. HIV-1 infected individuals were recruited from the outpatient clinic of the Peking Union Medical College Hospital, China.

### HIV-1 stocks

Calcium-phosphate-mediated transfection of HEK293T cells was used to generate virus stock. Pseudotyped single-cycle infectious HIV-Luc/VSV-G or HIV-Luc/NL-3 was obtained by cotransfection with the luciferase reporter HIV-1 proviral plasmid pLAI-Δ-env-Luc and the expression plasmid vesicular stomatitis virus G (VSV-G) protein or HIV-1-NL4-3 Env; replication competent HIV-1-NL4-3 (CXCR4 tropic) virus was generated by transfection with pNL4-3 of HIV-1 proviral vector as previously described (65). Harvested supernatants of transfected cells that contained viral particles were filtered and titrated with p24^gag^ capture enzyme-linked immunosorbent assay (ELISA). The HIV-1 p24^gag^ specific monoclonal antibodies were kindly provided by Prof. Yong-Tang Zheng (Kunming Institute of Zoology, Chinese Academy of Sciences, China). Viral infection was measured by detecting luciferase activity using the Luciferase assay system (Promega) or Real-time (RT-) PCR to detect viral *gag* mRNA expression.

### Plasmids

pcDNA3.1 plasmid containing lncRNA MALAT1 was purchased from Integrated Biotech Solutions (Shanghai, China). Luciferase-based reporter vector pGL3 plasmids containing China-B′, C and 07/08-BC subtypes of HIV-1 LTR were described previously (66). The HIV-1 Tat-expressing plasmid (pTat) was kindly provided by Dr Li Wu (The Ohio State University, USA).

### RNA extraction, library preparation and deep sequencing

Total RNAs were extracted from samples using TRIzol (Invitrogen), and DNA digestion was carried out with DNaseI. RNA Integrity was confirmed by 1.5% agarose gel electrophoresis. RNAs were quantified by Qubit 3.0 with QubitTM RNA Broad Range Assay kit (Life Technologies). A total of 2 μg of RNAs were used for stranded RNA sequencing library preparation. In brief, RNAs were iron-fragmented and used for first strand cDNA synthesis with random hexamers. The second strand cDNA was synthesized with RNase H, Klenow DNA polymerase and dNTPs, in which dTTP was replaced by dUTP. After end-repair and dA tailing, the double-stranded cDNAs were ligated to Illumina DNA P5 and P7 adapters. Prior to PCR amplification, the second strand cDNA was degraded by UDG to ensure strand specificity. PCR products corresponding to 200–500 bp were purified, quantified and finally sequenced on Hiseq X10 sequencer (Illumina).

### RNA-Seq data analysis

Raw sequencing data were first filtered by Trimmomatic (version: 0.36), low-quality reads were discarded and adaptor sequences were trimmed. Clean reads from each sample were mapped to the reference genome of Homo sapiens (Homo_sapiens. GRCh38; ftp://ftp.ensembl.org/pub/release-87/fasta/homo_sapiens/dna/) with default parameters. Reads mapped to the exon regions of each gene were counted by feature counts (Subread-1.5.1; Bioconductor) and the Reads Per kilobase per Million mapped read (RPKMs) were calculated. Genes differentially expressed between groups were identified using the edgeR package. A corrected *P*-value  cutoff of 0.05 and fold-change cutoff of 2 were used to determine statistically significant difference in gene expression. Gene ontology (GO) analysis of differentially expressed genes was performed using GO-seq R package, with a corrected *P*-value cutoff of 0.05 to judge statistically significant enrichment. Kyoto encyclopedia of genes and genomes (KEGG) enrichment analysis of DEGs was implemented by KOBAS software (version: 2.1.1).

### Construction of LentiCRISPR/Cas9 system

LentiCRISPR plasmid (pXPR_001) containing two expression cassettes, hSpCas9 and the chimeric guide RNA, were kindly provided by Dr Huan-Zhang Zhu (Fudan University, Shanghai, China). This vector enables lentiviral delivery of both Cas9 and sgRNA for targeted gene knockout. Two target sequences for the lncRNA MALAT1, 5′-CTG GTT CTA ACC GGC TCT AG-3′; 5′-CCT GAC GCA GCC CCA CCG GTT-3′, were cloned into the pXPR_001 plasmid. These target sequences were tested using the http://www.crispr.mit.edu/ tool to check for off-target effects. The scores showed that the designed targets had high quality guide. BLAST search against human whole genome confirmed the nonspecific binding of these designed gRNAs to other host genes. To package lentivirus, the lentiCRISPRs containing Cas9 and MALAT1 specific or off-target gRNAs were co-transfected into HEK293T cells with the expression plasmid of VSV-G protein and the lentiviral packaging plasmid psPAX2 (67). To generate cell lines with MALAT1 stable knockout, lentiviruses containing MALAT1 gRNA were used to infect HEK293T or Jurkat T cells. At 24 h post-infection, puromycin (1 μg/ml) was added for selection. Cells were then used for colony screening. Cell clones with MALAT1 stable knockout were confirmed with RT-PCR and sequencing.

### siRNA- and shRNA-mediated gene silencing

The sequences of siRNA duplexes were as follows: MALAT1 siRNA1, forward, 5′-GAG CAA AGG AAG UGG CUU ATT-3′ and reverse, 5′- UAA GCC ACU UCC UUU GCU CTT-3′. MALAT1 siRNA2, forward, 5′-GCG GAA GCU GAU CUC CAA UTT-3′ and reverse, 5′-AUU GGA GAU CAG CUU CCG CTT-3′. Off-target siRNA, forward, 5′-UUC UCC GAA CGU GUC ACG UTT-3′ and reverse, 5′-ACG UGA CAC GUU CGG AGA ATT-3′. siRNAs (4 nM) were transfected into HEK293T cells by using Lipofectamine 2000. The targeted sequences of shRNAs were as follows: MALAT1, 5′-AAG ACC TTG AAA TCC ATG ACG CTC GAG CGT CAT GGA TTT CAA GGT CTT-3′. EZH2, 5′-GCT AGG TTA ATT GGG ACC AAA CTC GAG TTT GGT CCC AAT TAA CCT AGC-3′. Off-target shRNA, 5′-TTC TCC GAA CGT GTC ACG TAT CTC GAG ATA CGT GAC ACG TTC GAG AA-3′. MALAT1 and EZH2 shRNA were subcloned into the pLKO.1-puro shRNA expression vector. Calcium phosphate-mediated transfection of HEK293T cells was used to generate shRNA lentiviruses as previously described (68).

### Real-time (RT) PCR analysis

Total cellular RNA was extracted with TRIzol reagent (Life Technologies) and then was reversely transcribed to cDNA with ReverTra Ace qPCR RT Master Mix with gDNA Remover Kit (TOYOBO). Genome DNA (gDNA) was extracted using QIAamp MiniElute DNA Kit (Qiagen) according to the user’s manual. RT-PCR was performed using the Thunderbird SYBR qPCR Mix (TOYOBO) on the ABI 7900HT Real-time PCR system, with an initial denaturation step for 10 min at 95°C, amplification with 40 cycles of denaturation (95°C, 30 s), annealing (55°C, 30 s) and extension (72°C, 30 s), followed by a final extension at 72°C for 6 min. The data were analyzed by SYBR green-based, semi-quantified method and normalized with GAPDH. The primers were used as follows: *GAPDH*, forward, 5′-GGG AAA TCG TGC GTG ACA T-3′ and reverse, 5′-GTC AGG CAG CTC GTA GCT CTT-3′. *Gag*, forward, 5′-GTG TGG AAA ATC TCT AGC AGT GG-3′ and reverse, 5′- CGC TCT CGC ACC CAT CTC-3′. Initial primers targeted base pair 10–59 of the HIV-1 transcript, forward, 5′-GTT AGA CCA GAT CTG AGC CT-3′ and reverse, 5′-GTG GGT TCC CTA GTT AGC CA-3′. Proximal primers targeted base pairs 29–180 of the HIV-1 transcript, forward, 5′-TGG GAG CTC TCT GGC TAA CT-3′ and reverse, 5′-TGC TAG AGA TTT TCC ACA CTG A-3′. Intermediate primers targeted base pair 836–1015 of the HIV-1 transcript, forward, 5′-GTA ATA CCC ATG TTT TCA GCA TTA TC-3′ and reverse, 5′-TCTGGCCTG GTG CAA TAGG-3′. Distal primers targeted base pair 2341–2433 of HIV-1 transcript, forward, 5′-GAG AAC TCA AGA TTT CTG GGA AG-3′ and reverse, 5′-AAA ATA TGC ATC GCC CAC AT-3′. Late RT primers, MH531, 5′-TGT GTG CCC GTC TGT TG TGT-3′ and MH532, 5′-GAGTCCTGCGTCGAGAGATC-3′; 2-LTR primers, MH535, 5′-AAC TAG GGA ACC CAC TGC TTA AG-3′, and MH536, 5′-TCC ACA GAT CAA GGA TAT CTT GTC-3′. *tat/rev*, forward, 5′-ATG GCA GGA AGA AGC GGA G-3′ and reverse, 5′-ATT CCT TCG GGC CTG TCG-3′.

### Antibodies

The following antibodies were used for Chromatin Immunoprecipitation (ChIP), RNA-binding protein Immunoprecipitation (RIP) or immunoblotting: anti-H3K27me3 (17–622, Millipore), anti-H3K9me3 (ab8898, Abcam), anti-EZH2 (07–689, Millipore), anti-SUZ12 (3737, Cell Signaling Technology), anti-EED (17–663, Millipore), anti-GAPDH (M20006, Abmart).

### Immunoblotting

Cells were lysed for 1 h at 4°C in ice-cold lysis buffer (50 mM 4-(2-hydroxyethyl)-1-piperazineethanesulfonic acid (HEPES), pH 7.4, 150 mM NaCl, 0.5 mM Ethylene Glycol Tetraacetic Acid (EGTA), 1% protease inhibitor cocktail [Sigma], 1 mM sodium orthovanadate, 1 mM NaF, 1% [vol/vol] Triton X-100 and 10% [vol/vol] glycerol). After centrifugation for 10 min at 12 000 *g*, the supernatant was boiled in reducing SDS sample loading buffer and analyzed by SDS-PAGE. For immunoblotting, the indicated specific primary antibodies were used, followed by horseradish peroxidase-conjugated goat anti-mouse IgG or goat anti-rabbit IgG (Sigma) as the secondary antibody.

### Chromatin immunoprecipitation (ChIP)

Jurkat T cells or HEK293T cells with or without MALAT1 knockout were infected with HIV/luc-VSV-G virus (1 ng p24^gagag^/1 × 10^6^) for 2 days. Cells were then cross-linked in 1% formaldehyde for 10 min at room temperature and quenched with 0.125 M glycine for 5 min. After lysis, chromatin was sheared by sonication for 12 min (10 s on and 10 s off) on ice to obtain DNA fragments of 200 to 1000 bp. About 5% of the total sheared chromatin DNA was used as input. Sheared chromatin was incubated with an antibody against EZH2, H3K27me3 or H3K9me3. Rabbit and mouse IgG were used as negative controls. The immunoprecipitated DNA was analyzed by real-time PCR (ABI Prism 7900 real-time PCR system) for 30 cycles with *Taq* master mix (Invitrogen). The primers targeting for HIV LTR Nuc0, DHS, Nuc1 and Nuc2 regions had been described previously (68–70). Nuc0, forward, 5′-TGG ATC TAC CAC ACA CAA GG-3′ and reverse, 5′-GTA CTA ACT TGA AGC ACC ATC C-3′. DHS, forward, 5′-AAG TTT GAC AGC CTC CTA GC-3′ and reverse, 5′-CAC ACC TCC CTG GAA AGT C-3′. Nuc1, forward, 5′-TCT CTG GCT AAC TAG GGA ACC-3′ and reverse, 5′-CTA AAA GGG TCT GAG GGA TCT C-3′. Nuc2, forward, 5′-AGA GAT GGG TGC GAG AGC-3′ and reverse, 5′-ATT AAC TGC GAA TCG TTC TAG C-3′.

### RNA-binding protein immunoprecipitation (RIP)

RNA-binding protein immunoprecipitation (RIP) was performed in a native condition. Briefly, Jurkat T cells were lysed and cell nuclei were isolated and suspended in 1 ml ice-cold RIP Buffer (150 mM KCl, 25 mM Tris, pH 7.4, 5 mM ethylenediaminetetraacetic acid, 0.5 mM Dithiothreitol (DTT), 0.5% NP40, 100 U/ml RNAase and protease inhibitor cocktail). The chromatin was sheared by ultra-sonication and centrifuged for 10 min to remove debris, followed by incubation for immunoprecipitation with specific antibodies against EZH2, SUZ12 or EED. 5% of each sample was used as input. Rabbit or mouse IgG was used as a negative antibody control. The immunoprecipitated RNA was extracted using Trizol (Invitrogen) and analyzed by real-time (RT-) PCR (ABI Prism 7900 real-time PCR system) for 40 cycles with Taq master mix (Invitrogen), using primers specific for the MALAT1 detection, forward, 5′-CTT CCC TAG GGG ATT TCA GG-3′ and reverse, 5′-GCC CAC AGG AAC AAG TCC TA-3′.

### Assays for HIV-1 reactivation

HIV-1 latently infected ACH2 or C11 clone cells were stimulated with PMA (phorbol-12-myristate-13-acetate)/Ionomycin or SAHA (Vorinostat) (Sigma) at indicated concentrations for 24 h. Viral reactivation was detected by titrating the produced viral particles in TZMB1 indicator cells by measuring luciferase activity, or quantifying the cell-associated *gag* mRNA, or measuring GFP expression in C11 cells. MALAT1 expression was semi-quantified by RT-PCR and normalized with GAPDH.

### Statistical analysis

Statistical analysis was performed using Wilcoxon signed-rank test.

## RESULTS

### RNA-Seq analysis reveals an up-regulation of MALAT1 upon HIV-1 infection

**Figure 1.**
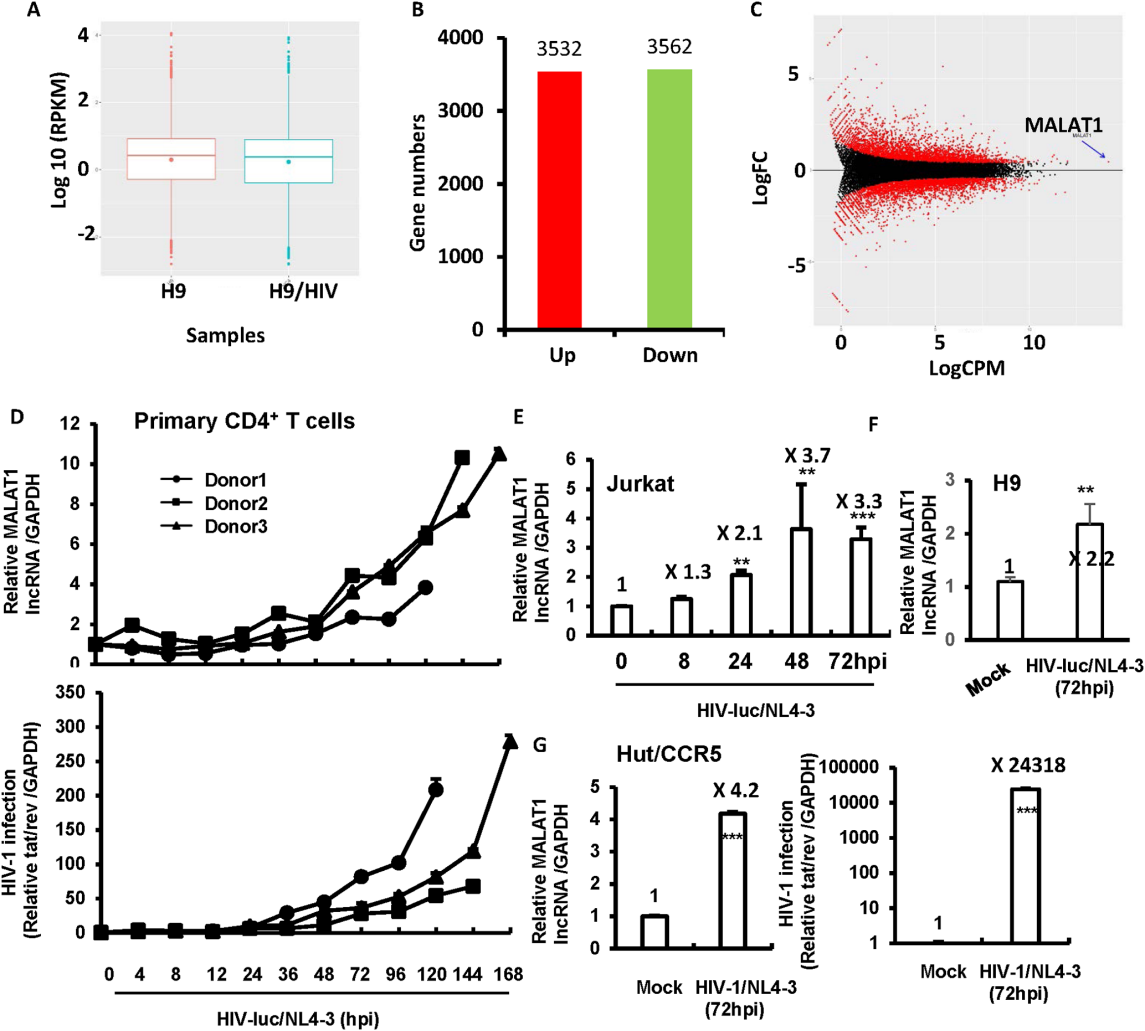
LncRNA profiles in HIV-1 NL4-3-infected and uninfected CD4^+^ T cells. (**A**) Overall gene expression in HIV-1 NL4-3-infected CD4^+^ T cells (H9) and uninfected counterpart. The lines inside each box indicate the median value of overall gene expression. The *y*-axis denotes the positive log (base 10) of RPKM. (**B**) Numbers of differentially expressed genes in HIV-1 NL4-3-infected H9 cells compared with uninfected controls. The *y*-axis denotes the count number of genes. (**C**) MA plot of differentially expressed genes in HIV-1 NL4-3-infected H9 cells. The *y*-axis indicates log (base 2) of the fold change (FC) for difference in expression. The *x*-axis indicates log (base 2) of the tags per million reads (TPM). The red spots indicate the differentially expressed genes, and the black line denotes the cutoff value (logFC = 0). The red arrowhead indicates MALAT1-encoding gene. (**D**–**G**) MALAT1 expression upon HIV-1 infection. PHA-P-activated primary CD4^+^ T cells (D), Jurkat T cells (E), H9 cells (F) and Hut/CCR5 cells (G) were infected with either pseudotyped HIV-Luc/NL-3 (D, E, F, 2 ng p24^gag^) or replication competent HIV-1/NL-3 (G, 2 ng p24^gag^), respectively, for indicated time. The endogenous expression of MALAT1 was quantified by RT-PCR, and HIV-1 infection was monitored by RT-PCR for quantifying the production of HIV-1 *tat/rev* mRNA. Hpi, hours post-infection. Data were expressed as mean ± SD. Results were representative of at least three independent experiments. **P<0.01 and ***P<0.001, were considered as significant differences as determined by an unpaired *t*-test.

We first examined whether HIV-1 can regulate the expression of MALAT1 by performing RNA-seq analysis in HIV-1-NL4-3-infected and uninfected CD4^+^ T cells (H9 cell), as described in ‘Materials and methods’ section. The overall gene expression level of two samples was similar (Figure 1A), suggesting that the rRNA depletion-based library preparation method has worked. The genes differentially expressed between HIV-1-NL4-3-infected and uninfected H9 cells were then identified using the edgeR package. Compared with uninfected H9 cells, the infected cells had 3532 and 3562 genes up-regulated or down-regulated in expression, respectively (Figure 1B). *MALAT1* was the most markedly up-regulated gene in HIV-1 NL4-3-infected cells, with the highest logCPM value (Figure 1C). *MALAT1* also displayed the nearly highest RPKM value of overall gene expression, among 1815.664 genes in uninfected cells and 2489.684 genes in HIV-1 NL4-3-infected cells. Only by the log2FC (0.46) calculation, a few genes had higher expression levels than *MALAT1*. We have deposited the corresponding raw data in the GEO library. The GEO number is GSE124466.

To confirm that the up-regulation of MALAT1 upon HIV-1 infection is universal, we also used PHA-P-activated primary CD4^+^ T cells, CD4^+^ T-lymphocyte cell line Hut/CCR5, Jurkat and H9 cells for infection with either pseudotyped HIV-Luc/NL4-3 or replication competent HIV-1/NL4-3. The time-course of infection (Figure 1D and E) and the 72-h infection (Figure 1F and G) by HIV-1 significantly increased MALAT1 expression in all these CD4^+^ T-lymphocyte cell types. Taken together, these results demonstrate that MALAT1 expression is up-regulated upon HIV-1 infection.

### MALAT1 expression is required for promoting optimal HIV-1 infection

**Figure 2.**
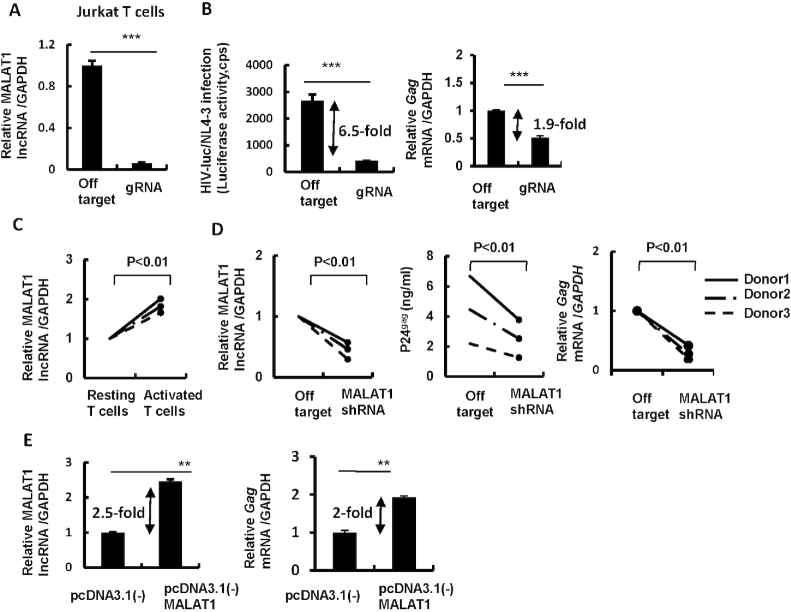
MALAT1 activates HIV-1 infection by enhancing viral transcription. (**A** and **B**) MALAT1 knockout decreases HIV-1 infection and transcription in Jurkat T cells. MALAT1-stably-knocking-out Jurkat T cells were infected with pseudotyped HIV-luc/NL4-3, MALAT1 knockout was identified with RT-PCR (A). Viral infection was measured 3 days after infection by detecting luciferase activity and quantifying transcriptional levels of *gag* mRNA (B). (**C**) MALAT1 expression in primary CD4^+^ T cells. Resting CD4^+^ T cells (1 × 10^6^) from healthy donors were stimulated with or without PHA-P (5 μg/ml) for 3 days, then cells were harvested and the endogenous expression of MALAT1 was quantified by RT-PCR. (**D**) MALAT1 knockout decreases HIV-1 replication and transcription. PHA-P-activated CD4^+^ T cells were infected with lentivirus containing MALAT1-specific shRNA or off-target control for 2 days, and then infected with the replication competent virus HIV-1/NL4-3 (2 ng p24^gag^) for an additional 4 days. The knockout of MALAT1 was monitored with RT-PCR at 4 days post infection; viral production was detected by either quantifying p24^gag^ levels in the supernatants using ELISA or quantifying cell-associated HIV-1 gag mRNA using RT-PCR. (**E**) MALAT1 overexpression inhibits HIV-1 infection. PHA-P-activated CD4^+^ T cells were nucleofected with pcDNA3.1(-) MALAT1 (or vector control) for 48 h, then cells were infected with HIV-Luc/NL4-3 for an additional 2 days. The expression of MALAT1 and cell-associated HIV-1 *gag* mRNA were quantified with RT-PCR. Data were presented as mean ± SD. Results were representative of at least three independent experiments (A, B, E). **P<0.01 and ***P<0.001 were considered as significant difference as determined by an unpaired *t*-test.

To determine the specific role of MALAT1 in HIV-1 infection, we knocked the *MALAT1* gene in Jurkat CD4^+^ T cells by transduction with lentiviruses containing *MALAT1*-specific lentiCRISPR gRNAs that targeting five nucleotides in the *MALAT1* promoter for deletion, as confirmed by sequencing (Supplementary Figure S1A), and then infected these cells with HIV-Luc/NL4-3 virus for additional 48 h. Results showed that the MALAT1 knockout significantly impaired HIV-1 infection (Figure 2A and B, left panel).

Further mechanistic analyses revealed that MALAT1 affected HIV-1 life-cycle at post-integrational steps, because HIV-1 reverse transcription, nuclear entry and integration as quantified with Late-RT, 2-LTR and *gag*-PCR, respectively, showed similar levels in MALAT1 knockout and off-target control cells (Supplementary Figure S2A and C). However, the production of HIV-1 *gag* mRNA was significantly decreased in MALAT1 knockout cells (Figure 2B, right panel), suggesting that MALAT1 works at promoting the transcription of HIV-1 proviral DNA.

To confirm these findings, we used specific small interfering RNAs (siRNAs) to knockdown endogenous MALAT1 in HEK293T cells (Supplementary Figure S3A), and observed significantly diminished infection of a single-cycle infectious HIV-Luc/VSV-G virus (Supplementary Figure S3B), and significantly decreased expression of *gag* mRNA (Supplementary Figure S3C). HIV-1 reverse transcription, nuclear entry and integration as quantified with Late-RT, 2-LTR and *gag*-PCR, respectively, showed similar levels in MALAT1 knockdown and off-target control cells (Supplementary Figure S3D and F), confirming MALAT1 promotes HIV-1 transcription.

To examine whether the same mechanisms are in operation in primary cells, we stimulated primary CD4^+^ T cells and with PHA-P significantly and found elevated MALAT1 expression in resting primary CD4^+^ T cells isolated from healthy donors (Figure 2C). We then knocked down MALAT1 expression in PHA-P-activated primary CD4^+^ T cells by infection with lentiviruses containing MALAT1 shRNA for 2 days (Figure 2D, left panel), and then infected them with replication competent virus HIV-1/NL4-3 for additional 4 days. Results showed that MALAT1 knockdown significantly reduced viral particles released into the supernatant as quantified by p24^Gag^ production (Figure 2D, middle panel); in parallel, the expression of HIV-1 *gag* mRNA was also significantly reduced (Figure 2D, right panel), confirming MALAT1 is required for optimal HIV-1 infection and replication.

To further confirm the role of MALAT1 in HIV-1 infection, we overexpressed it by transfecting the pcDNA3.1/MALAT1 into PHA-P-activated primary CD4^+^ T cells, and then infected these cells with HIV-Luc/NL4-3. Results showed that viral infection was significantly increased as measured by the production of *gag* mRNA (Figure 2E). Taken together, these results demonstrate that MALAT1 promotes HIV-1 infection.

### MALAT1 expression initiates HIV-LTR-driven gene expression

**Figure 3.**
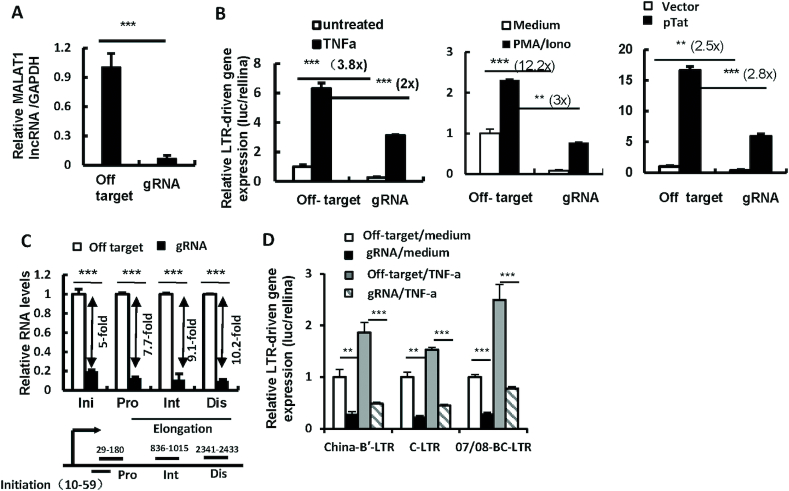
MALAT1 promotes HIV-1 LTR-driven gene expression. (**A** and **B**) MALAT1 knockout significantly inhibits HIV 5′-LTR-driven transcription. MALAT1-stably-knocking-out HEK293T cells were co-transfected with pGL3-LTR-luc plasmid that contains an HIV-1NL4-3-LTR promoter driven luciferase reporter, and pRenilla-luc-TK (inner control) for 24 h. Then cells were treated with TNF-α (50 ng/ml) or PMA (20 nM) /Ionomycin (1.5 μM) or transfected with pTat for an additional 24 h. MALAT1 knockout was monitored with RT-PCR (A), and the reporter gene expression was assessed by luciferase assay (B). (**C**) MALAT1 knockout decreases HIV 5′-LTR-driven transcription initiation and elongation. MALAT1-stably-knocking-out Jurkat T cells were infected with HIV-luc/VSV-G for 2 days and total mRNAs were extracted. The HIV-1 gene transcription initiation and elongation were assessed by qPCR with specific primers. (**D**) MALAT1 knockout represses LTR-driven gene expression. MALAT1-stably-knocking-out HEK293T cells were co-transfected with different subtypes of HIV-1 LTR and pRenilla-luc-TK for 24 h, cells were then treated with TNF-α (50 ng/ml) for an additional 24 h. The reporter gene expression was assessed by luciferase assay. Data were presented as mean ± SD. Results were representative of at least three independent experiments. **P<0.01 and ***P<0.001 were considered as significant difference as determined by an unpaired *t*-test.

HIV-1 LTR promoter plays an essential role in driving viral transcription and productive infection (71–73). Having demonstrated the important role of MALAT1 expression for HIV-1 transcription, we next investigated whether MALAT1 can regulate LTR activity. The endogenous MALAT1 in HEK293T cells could be efficiently knocked out by infection with lentiviruses containing MALAT1 specific lentiCRISPR gRNAs (or off-target control) (Supplementary Figure S1B and Figure 3A). These cells were then transfected with a luciferase reporter driven by the full-length LTR promoter from HIV-1 NL4-3 and treated with or without TNF-α or PMA/Ionomycin cocktail to induce gene expression. We observed that MALAT1 knockout significantly impaired both basal- and stimulated LTR-driven gene expression (Figure 3B, left and middle panels). Because HIV-1 Tat protein binds to *trans*-activation response element to drive transcription elongation, we next used this to examine the effect of MALAT1. Results showed that in cells treated with pTat, the LTR-driven gene expression was also significantly reduced in MALAT1 knockout cells (Figure 3B, right panel). These data further demonstrate that MALAT1 increases HIV-1 LTR-driven gene expression.

The initiation and elongation of HIV-LTR-driven transcription can be assessed by quantifying the production of various lengths of viral messenger RNAs with (RT-) PCR using specific primers (68,70). We found that MALAT1 knockout significantly decreased the production of initial viral message RNAs, but did not further decrease the production of elongated viral RNAs, indicating that the depletion of MALAT1 impaired the initiation of HIV-LTR-driven transcription, but not the elongation (Figure 3C). The same result was also observed in HEK293T cells when MALAT1 was knockdown with small interfering RNAs (Supplementary Figure S3G). In addition to laboratory adapted HIV strains, we further investigated the role of MALAT1 on clinical HIV isolates by cloning LTRs from Chinese HIV-1 subtypes B′, CRF07/08_BC and C (66), and found the same impairment on all these LTR-driven gene expressions when MALAT1 was knockedout (Figure 3D). Taken together, these data further demonstrate that MALAT1 promotes HIV-LTR-driven transcription as a universal phenomenon.

### MALAT1 associates with PRC2 and prevents HIV-1 5′-LTR from being trimethylated at histone H3 on lysine 27

MALAT1 has been reported to bind PRC2 complex in cancer cells (37,53). PRC2 primarily trimethylates H3K27me3 to silence gene transcription (74,75), and its catalytic subunit EZH2 is known to facilitate H3K27 trimethylation on LTR of HIV-1 provirus to silence viral transcription, and thus contributes to the establishment and maintenance of viral latency (56–60). Based on these observations, we hypothesize that MALAT1 promotes HIV-1 replication by antagonizing the inhibitory role of PRC2.

**Figure 4.**
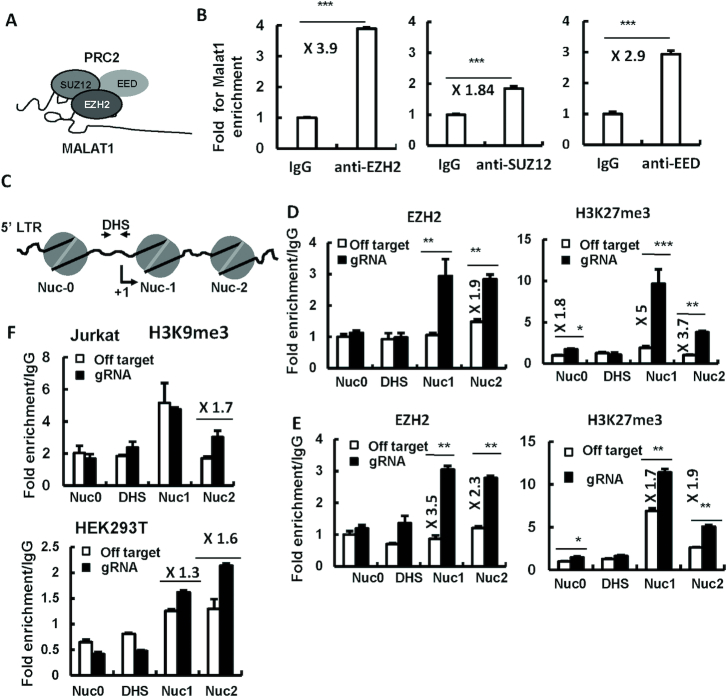
MALAT1 knockout promotes EZH2 binding to HIV-1 LTR and increases the modification of H3K27me3. (**A**) A schematic illustration of the interaction between MALAT1 and PRC2 subunits. (**B**) Associations of EZH2, SUZ12 and EED with MALAT1 in Jurkat T cells as determined with RIP assay. (**C**) A schematic illustration of nucleosomes on HIV-1 5′-LTR region. (**D**–**F**) MALAT1 knockout promotes EZH2 binding to HIV-1 LTR and increases the modification of H3K27me3. MALAT1-stably-knocking-out HEK293T (D) or Jurkat T cells (E), were infected with HIV-luc/VSV-G for 2 days, the associations of EZH2 (D, E, left panels), H3K27me3 (D, E, right panels) and H3K9me3 (F) with HIV-1 5′-LTR were determined by a cross-linked ChIP assay. Data were presented as mean ± SD. Results were representative of at least three independent experiments. **P < 0.01 and ***P < 0.001 denote significant differences as determined by an unpaired *t*-test.

To prove this hypothesis, we first verified the association of MALAT1 with PRC2 complex through RIP assay. In Jurkat CD4^+^ T cells, MALAT1 showed interaction with the three core components of PRC2 complex: EZH2, SUZ12 and EED (Figure 4A and B). The 5′-LTR of HIV-1 proviral DNA is organized into three strictly positioned nucleosomes (Nuc-0, Nuc-1 and Nuc-2), separated by two intervening enhancer regions DHS (DNase hypersensitive site)-1 and DHS-2 (Figure 4C). Nuc-1 is positioned immediately downstream of the LTR transcription start site and its disruption is required for LTR activation (76). To investigate the effect of MALAT1 on the methylation of HIV-1 LTR, we performed ChIP analysis in MALAT1-stably-knockout cells, and observed an increased recruitment of EZH2 to HIV-1 5′-LTR Nuc1 and Nuc2 regions after MALAT1 being knockedout in either HEK293T (Figure 4D, left panel) or Jurkat CD4^+^ T cells (Figure 4E, left panel). Consequently, HIV-1 5′-LTR Nuc1 and Nuc2 regions showed increased H3K27me3 levels in knockout cells than that in control cells (Figure 4D and E, right panels). The specificity of MALAT1 on PRC2-mediated H3K27me3 was also demonstrated a minor change of H3K9me3 levels (from no change to up to 1.7-fold enhancement) at the HIV-1 5′-LTR Nuc1 and Nuc2 regions after MALAT1 being knockout in either Jurkat CD4^+^ T (Figure 4F, upper panel) or HEK293T cells (Figure 4F, bottom panel). Taken together, these data prove that MALAT1 prevents the association of EZH2 with HIV 5′-LTR and reduces the epigenetic modification of LTR by PRC2.

### MALAT1 promotes HIV-1 infection by antagonizing EZH2-mediated silencing of viral gene transcription

**Figure 5.**
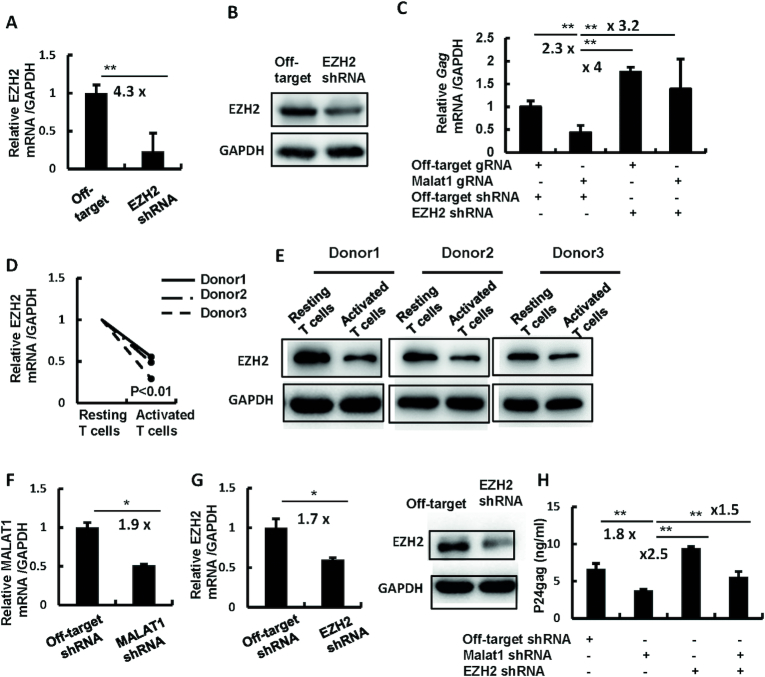
MALAT1 antagonizes EZH2-mediated silencing of viral gene transcription to promote HIV-1 replication. (**A–C**) MALAT1 restores HIV-1 infection by antagonizing EZH2-mediated inhibition. MALAT1-stably-knocking-out HEK293T cells were infected with lentivirus containing EZH2 specific shRNA or off-target controls for 48 h to further knockdown EZH2 expression, cells were then infected with HIV-luc/VSV-G for an additional 24 h. EZH2 knockdown was detected by RT-PCR (A) and western blot (B). Viral infection was detected by quantifying cell-associated HIV-1 *gag* mRNA (C). (**D** and **E**) EZH2 expression in primary CD4^+^ T cells. Resting CD4^+^ T cells (1 × 10^6^) were stimulated with or without PHA-P (5 μg/ml) for 3 days, and the endogenous expression of EZH2 was detected by either RT-PCR (D) or western blot (E). (**F**–**H**) The double knockdown of MALAT1 and EZH2 rescues HIV-1 infection in primary CD4^+^ T cells. PHA-P-activated primary CD4^+^ T cells (1 × 10^6^) were infected with lentiviruses containing MALAT1 or/and EZH2-specific shRNA or off-target controls for 48 h, then cells were infected with replication-competent HIV-1 NL4-3 for an additional 96 h. MALAT1 knockdown was determined by RT-PCR (F); the expression of endogenous EZH2 was detected by RT-PCR and western blot (G); viral replication was quantified by detecting p24^gag^ levels in the supernatants by ELISA (H). Data were presented as mean ± SD. Results were representative of three independent experiments. *P < 0.05 and **P < 0.01 denote significant differences as determined by an unpaired *t*-test.

We have above demonstrated that MALAT1 detached EZH2 from HIV-1 LTR and reduces the repressive H3K27me3 of LTR regions. To investigate whether MALAT1-promoted HIV-1 infection was due to the antagonism of EZH2-mediated silencing of viral gene transcription, we further knocked down EZH2 expression with specific shRNA in MALAT1-stably-knocking-out HEK293T cells (Figure 5A and B), and then infected cells with HIV-luc/VSV-G for an additional 24 h. These manipulation of cell gene expression led to a recovery of HIV-1 transcription as quantified by the production of cell-associated HIV-1 *gag* mRNA (Figure 5C).

To further confirm this result, we performed the same assay using primary CD4^+^ T cells. EZH2 showed high expression level in resting CD4^+^ T cells isolated from healthy donors but diminished expression upon activation with PHA-P, as displayed at both mRNA and protein expression levels (Figure 5D and E). By transduction with lentivirus containing specific shRNAs, the mRNA and/or protein expression levels of MALAT1 and EZH2 decreased in PHA-P-activated primary CD4^+^ T cells (Figure 5F and G). The double knockdown of MALAT1 and EZH2 resulted in rescued replication of HIV-1 NL4-3 (Figure 5H). These results demonstrate that MALAT1 promotes HIV-1 replication by antagonizing EZH2-mediated silencing of viral gene transcription.

### HIV latency reversal agents (LRAs) induce MALAT1 expression in latently infected cells

**Figure 6.**
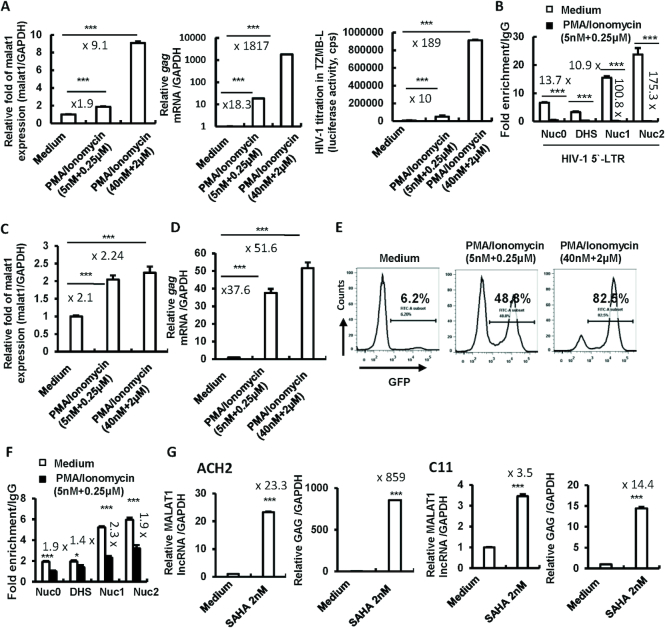
LRAs induce MALAT1 expression in HIV-1 latently infected cells. HIV-1 latently infected CD4^+^ CEM cells (ACH2) (**A**) and Jurkat T-cell (C11 clone) (**C–E**) were stimulated with PMA/Ionomycin at indicated concentrations for 24 h, and viral reactivation was detected by quantifying the cell-associated *gag* mRNA (A, middle panel; D), or titrating the produced viral particles in TZMB1 indicator cells (A, right panel), or detecting GFP expression (E). The associations of EZH2 with HIV-1 5′-LTR were determined by a cross-linked ChIP assay (B and **F**). (**G**) MALAT1 expression by stimulation with SAHA. ACH2 or C11 were treated with SAHA for 24 h, viral reactivation was detected by quantifying cell-associated HIV-1 *gag* mRNA. MALAT1 expression was quantified by RT-PCR and normalized with GAPDH (A, C and G). Results were representative of three independent experiments. *P < 0.05 and ***P < 0.001 denote significant differences as determined by an unpaired t-test.

The reversible silencing of LTR-driven transcription is critical for an integrated provirus to maintain viral latency (68,70,77–80). The promotion effect of MALAT1 on HIV-1 LTR-driven gene expression suggests a potential role for MALAT1 in reactivating HIV-1 from latency. Therefore, we investigated whether there is an association between MALAT1 expression and HIV reactivation. ACH2 cell is derived from HIV-1 latently infected CD4^+^ CEM cells that contain a single copy of proviral DNA per cell, and a point mutation in Tat response element within the LTR region, and thus an impaired Tat-mediated viral reactivation; but viruses can still be reactivated from ACH2 cells to produce infectious HIV-1 particles by stimulation with other reagents (62–64). Upon stimulation with PMA/Ionomycin, HIV-1 was reactivated from ACH2 cells, as demonstrated by HIV-1 *gag* mRNA production or infectious virus titrated in the TZMB1 indicator cells; accompanying HIV-1 reactivation, the endogenous expression of MALAT1 also showed remarkable elevation (Figure 6A), and the EZH2 recruitment to HIV-1 5′-LTR regions was markedly reduced (Figure 6B).

To confirm that the observed correlation of MALAT1 expression and HIV-1 reactivation is not an artifact in a single cell line, we tested this in the HIV-1 latently infected Jurkat T-cell (C11 clone) that harboring an HIV-1 proviral DNA encoding GFP (68,70,77,81). Similar to results obtained in ACH2 cells, upon stimulation with PMA/Ionomycin (Figure 6C), the endogenous expression of MALAT1 was elevated (Figure 6C); accompanying the elevated MALAT1 expression, HIV-1 was reactivated from C11 clone, as demonstrated by HIV-1 *gag* mRNA production (Figure 6D), and GFP expression (Figure 6E), and the reduction of EZH2 recruitment to HIV-1 5′-LTR regions was observed (Figure 6F). Additionally, the treatment with other LRA such as SAHA (Vorinostat) could increase MALAT1 expression in both ACH2 and C11 clone cells (Figure 6G). These data demonstrate a positive association between MALAT1 expression and HIV reactivation.

### Successful cART treatment is accompanied by decreased MALAT1 expression

**Figure 7.**
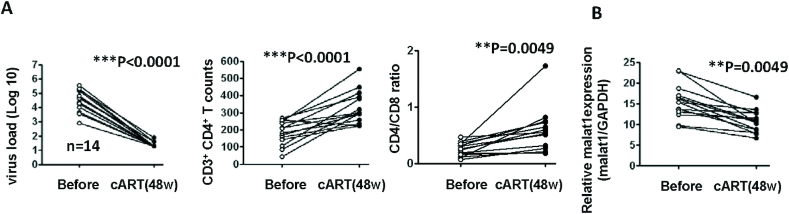
Successful cART treatment decreases MALAT1 expression. PBMCs were collected from HIV-1 infected individuals at pre-therapy and 48 weeks of cART-therapy time points. (**A**) Virus load, CD3^+^ CD4^+^ T counts, and CD4/CD8 ratio were analyzed. (**B**) MALAT1 expression was quantified with RT-PCR and normalized with GAPDH. **P <0.01 and ***P <0.001 denote significant differences as determined by an unpaired *t*-test.

To examine the clinical association between HIV-1 replication on MALAT1 expression, we longitudinally collected PBMCs from HIV-1 infected patients at pre-therapy and 48 weeks post cART-therapy time points. The 48-weeks cART therapy successfully suppressed plasma viral load to below the level of detection (<50 copies), increased CD4^+^ T counts, and restored CD4/CD8 ratio in 14 patients (Figure 7A). This was accompanied by significantly diminished MALAT1 expression (Figure 7B). These data directly linked the inhibition of HIV-1 replication to a reduced MALAT1 expression.

## DISCUSSION

**Figure 8.**
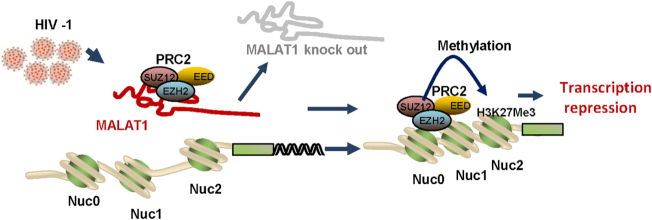
A schematic illustration of MALAT1 activates HIV-1 replication by displacing PRC2 from binding to the LTR and preventing it from mediating epigenetic silencing. The association of MALAT1 with PRC2 detaches the core component EZH2 from binding to HIV-1 LTR promoter, thus preventing epigenetic silencing caused by EZH2-mediated H3K27me3 methylation of HIV-1 LTR regions.

NcRNAs are implicated in a wide variety of cellular processes, and accumulating evidences have suggested ncRNAs including lncRNAs regulate HIV-1 infection (26,29,82). In this study, we discovered that lncRNA MALAT1 expression is required for efficient HIV-1 infection. Mechanistically, through binding to PRC2, MALAT1 prevents EZH2, a core component of PRC2, from binding to HIV-1 LTR promoter, making EZH2 unable to mediate methylation of LTR and epigenetic silencing of gene transcription (Figure 8).

By targeting distinct cellular machineries or viral components, lncRNAs could either repress or activate HIV-1 replication. Previously known lncRNAs 7SK RNA, NEAT1, NRON repress HIV-1 infection (14–20,24–26,28–30), and lncRNAs uc002yug.2 activates HIV-1 replication and reactivates HIV-1 from latency (31). Our study has added lncRNA MALA1 to the list of activators of HIV-1 infection.

At the molecular level, however, it is not entirely clear how lncRNAs modulate HIV-1 replication. Nucleus-located lncRNAs can influence chromatin architecture by interacting with chromatin-modulating proteins, such as Switch/sucrose nonfermentable (SWI/SNF) or PRC subunits, regulating their recruitment or association with chromatin, thereby influencing the association of specific transcription factors or other chromatin modulators to specific gene loci (12). Mammalian PRC2 can bind thousands of RNAs *in vivo* and be recruited by lncRNAs to specific sites of the promoter region, and thus epigenetically silence the expression of certain genes (83). PRC2 primarily trimethylates H3K27me3 to silence gene transcription (74,75). The catalytic subunit EZH2 facilitates H3K27 trimethylation on LTR of HIV-1 provirus to silence viral transcription and maintain viral latency (56–60).

In HIV-1 infected cells, we observed an association between MALAT1 and PRC2 subunits. But unlike in cancer cells where MALAT1 binding facilitates PRC2 to be associated with the promoter of specific gene locus (53–55), in HIV infection, the binding of MALAT1 with PRC2 displaced its catalytic component EZH2 from binding to HIV-1 LTR promoter, and thus preventing the HIV-1 LTR from PRC2-mediated epigenetic silencing. The differential interactions between PRC2 and specific promoters as modulated by MALAT1 may be a reflection of distinct cellular environments in which such modulation occurs.

The reversible silencing of HIV-1 LTR-driven transcription is the key for an integrated provirus to maintain viral latency (78–80). Given the critical role of MALAT1 in promoting HIV-1 LTR-driven transcription, we also investigated the correlation between MALAT1 expression and HIV-1 reactivation from latency. Indeed, the reactivation of HIV-1 by latency-reversing agents PMA/Ionomycin or SAHA both elevated the expression of MALAT1.

MALAT1 expression is upregulated in various cancers and thus has been proposed as a prognostic biomarker of metastasis (5,32–37). In HIV-1 infected individuals, we confirmed that successful cART-treatment could significantly reduce expression of MALAT1 (61). Whether MALAT1 could be a biomarker in HIV-1 infection is an interesting topic for future studies.

Taken together, we have identified the critical role of lncRNA MALAT1 in promoting HIV-1 transcription and infection at molecular levels. Our findings may provide a new therapeutic target for combating HIV infection.

## DATA AVAILABILITY

The data have been deposited in GEO under accession number GSE124466 (https://www.ncbi.nlm.nih.gov/geo/).

## Supplementary Material

Supplementary Data
