# Supplementary material for: Long noncoding RNA MALAT1 releases epigenetic silencing of HIV-1 replication by displacing the polycomb repressive complex 2 from binding to the LTR promoter
Source: Nucleic Acids Res. 2019 Feb 21;47(6):3013–27. doi: 10.1093/nar/gkz117 (PMC6451131; doi:10.1093/nar/gkz117)
Supplement: Supplementary Data [file gkz117_supplemental_file.pdf]

Long noncoding RNA MALAT1 releases epigenetic silencing of HIV-1 replication by displacing the polycomb repressive complex 2 from binding to the LTR promoter

Di Qu, Wei-Wei Sun, Li Li, Li Ma, Li Sun, Xia Jin, Taisheng Li, Wei Hou, Jian-Hua Wang

Figure legends

Figure S1. gRNAs targeting MALAT1. gRNA1 (**A**) and gRNA2 (**B**) targeting *MALAT1* promoter were designed and the sequences were denoted. gRNAs were cloned into lentiCRISPR vectors and these packaged lentiviruses containing *MALAT1*-specific gRNAs were used to infect Jurkat T cells or HEK293T cells. Single cell clone with MALAT1 knockout were picked and proliferated. PCR fragment analysis showed 5 bases depletion in gRNA1 in Jurkat T cells (A) and 4 bases depletion in gRNA2 in HEK293T cells (B).

Figure S2. The effect of MALAT1-knockout on HIV-1 infection. MALAT1-stably-knocking-out Jurkat T cells were infected with HIV-Luc/NL4-3 for 24 h, and genome DNA was extracted. The products of late-RT, 2-LTR and HIV-1 *gag* DNA were quantified with PCR assay, respectively. Data are mean ± SD. Results were representative of three independent experiments.

Figure S3. MALAT1-knockdown represses HIV-1 transcription. HEK293T cells were transfected with MALAT1 specific siRNA or off-target for 24h, and cells were infected with HIV-luc/VSV-G for additional 24h. (**A**) MALAT1 expression was detected by RT-PCR. Viral infection was measured by detecting luciferase activity (**B**) or quantifying the levels of HIV-1 gag mRNA (**C**). Cellar DNA was isolated and the

products of late-RT (**D**), 2-LTR (**E**) and HIV-1 *gag* DNA (**F**) were quantified with PCR assay, respectively. (**G**) Total cellular mRNAs were extracted and HIV-1 transcription initiation and elongation were assessed by RT-PCR with specific primers. Data are mean ± SD. Results were representative of three independent experiments. *$p<0.05$ and **$p<0.01$were considered as significant difference in an unpaired *t*-test.

Figure S1

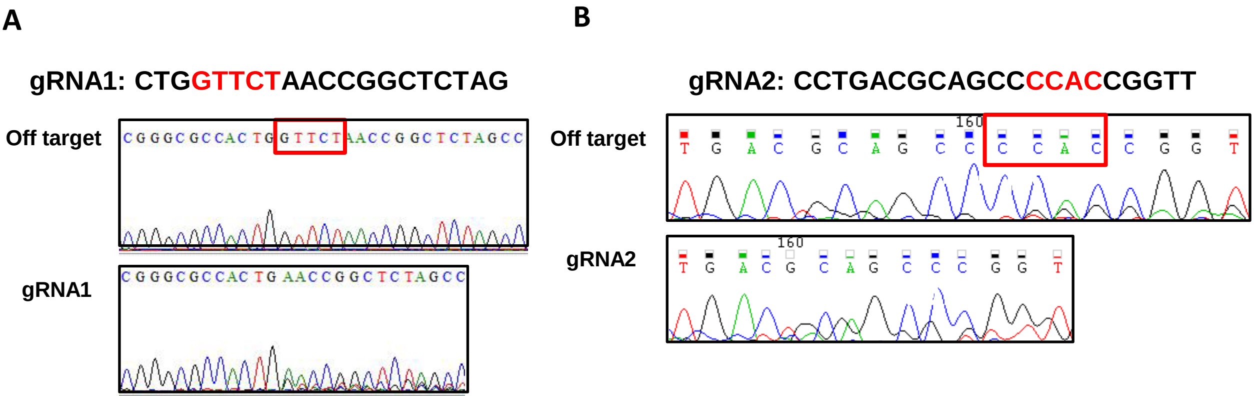
A
gRNA1: CTGGTTCTAACCGGCTCTAG
Off target
CGGGCGCCACTGGTTCTAACCGGCTCTAGCC
gRNA1
CGGGCGCCACTGAACCGGCTCTAGCC
B
gRNA2: CCTGACGCAGCCCCACCGGTT
Off target
160
T G A C G C A G C C C C A C C G G T
gRNA2
160
T G A C G C A G C C C G G T

Figure S2

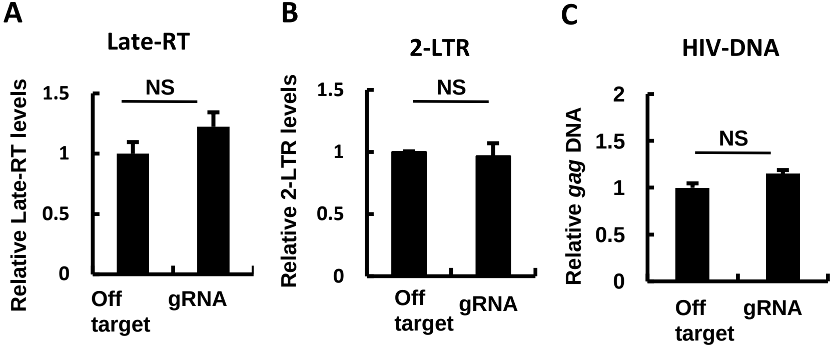

A
Late-RT
Relative Late-RT levels
1.5
1
0.5
0
NS
Off target
gRNA
B
2-LTR
Relative 2-LTR levels
1.5
1
0.5
0
NS
Off target
gRNA
C
HIV-DNA
Relative gag DNA
2
1.5
1
0.5
0
NS
Off target
gRNA

Figure S3

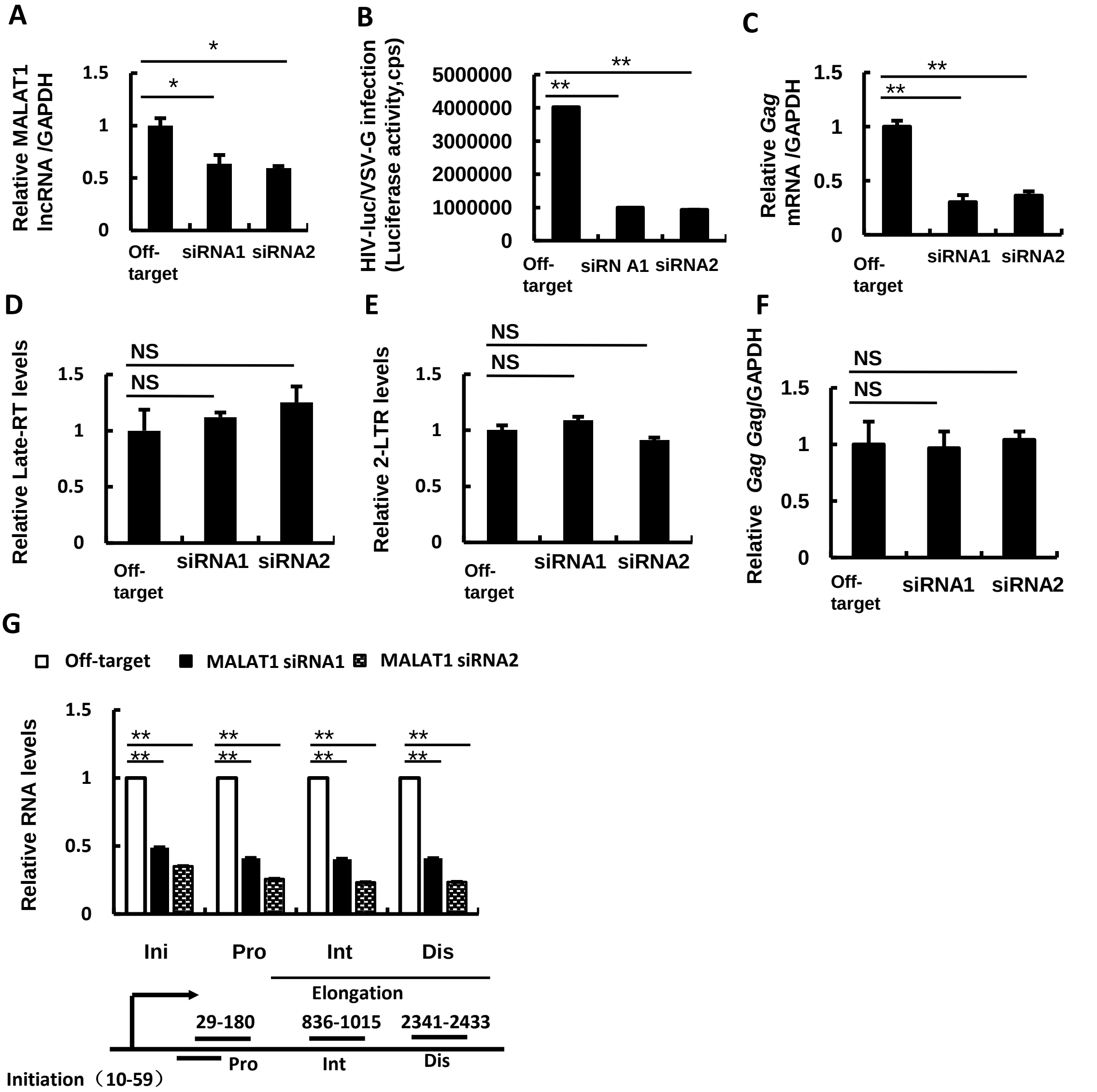

A
Relative MALAT1 lncRNA /GAPDH
1.5
1
0.5
0
*
*
Off-target
siRNA1
siRNA2
B
HIV-luc/VSV-G infection (Luciferase activity,cps)
5000000
4000000
3000000
2000000
1000000
0
**
**
Off-target
siRN A1
siRNA2
C
Relative Gag mRNA /GAPDH
1.5
1
0.5
0
**
**
Off-target
siRNA1
siRNA2
D
Relative Late-RT levels
NS
NS
1.5
1
0.5
0
Off-target
siRNA1
siRNA2
E
Relative 2-LTR levels
NS
NS
1.5
1
0.5
0
Off-target
siRNA1
siRNA2
F
Relative Gag Gag/GAPDH
NS
NS
1.5
1
0.5
0
Off-target
siRNA1
siRNA2
G
Off-target
MALAT1 siRNA1
MALAT1 siRNA2
Relative RNA levels
1.5
1
0.5
0
**
**
Ini
Pro
Int
Dis
Elongation
29-180
836-1015
2341-2433
Pro
Int
Dis
Initiation（10-59）
